# Induction of innate immunity and its perturbation by influenza viruses

**DOI:** 10.1007/s13238-015-0191-z

**Published:** 2015-07-24

**Authors:** Mohsan Ullah Goraya, Song Wang, Muhammad Munir, Ji-Long Chen

**Affiliations:** College of Animal Science, Fujian Agriculture and Forestry University, Fuzhou, 350002 China; The Pirbright Institute, Ash Road, Pirbright, Woking, GU24 0NF UK; CAS Key Laboratory of Pathogenic Microbiology and Immunology, Institute of Microbiology, Chinese Academy of Sciences, Beijing, 100101 China

**Keywords:** influenza A virus, innate immunity, immune escape

## Abstract

Influenza A viruses (IAV) are highly contagious pathogens causing dreadful losses to human and animal, around the globe. IAVs first interact with the host through epithelial cells, and the viral RNA containing a 5′-triphosphate group is thought to be the critical trigger for activation of effective innate immunity via pattern recognition receptors-dependent signaling pathways. These induced immune responses establish the antiviral state of the host for effective suppression of viral replication and enhancing viral clearance. However, IAVs have evolved a variety of mechanisms by which they can invade host cells, circumvent the host immune responses, and use the machineries of host cells to synthesize and transport their own components, which help them to establish a successful infection and replication. In this review, we will highlight the molecular mechanisms of how IAV infection stimulates the host innate immune system and strategies by which IAV evades host responses.

## INTRODUCTION

Influenza A viruses (IAV) cause highly pathogenic respiratory problems in human and animals. It is the major cause of annual epidemics and occasional pandemics in humans, responsible for 3–5 million cases of severe clinical infections and 250,000 to 500,000 fatal cases every year throughout the world (Stohr, 2002). Infection with influenza virus varies from subclinical infection of upper respiratory tract to lethal infection of lower respiratory system. Due to differences in pathogenesis of various influenza viruses, biology of the disease is not fully understood. Influenza virus infection induces host innate immune responses, which results in the termination of virus replication. On the other hand, IAV has evolved multiple strategies to circumvent the host innate immunity to establish a successful infection and replication.

In addition to typical seasonal infections, IAV can also undergo substantial changes (recombination/antigenic shift) that leave imprints of infection with little to no protective immunity and uplift mortality rate even among healthy young adults infected with IAV (Horimoto and Kawaoka, 2001; Palese, 2004). Only, the last century we have observed three major pandemics: the 1918 Spanish flu, 1957 Asian flu, and the 1968 Hong Kong flu; with the 1918 pandemic being the most concerning and significant, causing an estimated 30–50 million deaths worldwide (Horimoto and Kawaoka, 2001, 2005). Furthermore, the recent appearance of IAV strains with pandemic potential, such as H1N1 “swine flu” and H5N1 avian influenza, have highlighted the importance of studies about IAV infections and the innate and adaptive immune responses that control and eliminate infection. Thus, this review will focus on discussion of host innate immune responses to IAV infection and viral escape from the innate sensing.

## BIOLOGY AND STRUCTURE OF IAV

Influenza viruses are categorized in the family of *Orthomyxoviridae*. The virus particle is enveloped and contains a segmented, single-stranded, negative sense RNA genome (Klenk et al., 2004). Its genome possesses eight segments encoding 13 proteins (Jagger et al., 2012), out of which 8 are considered core proteins whereas the rest are called accessory proteins. Additionally, a putative open reading frame (ORF) in the positive-sense of segment 8 has been identified, which encodes for a hypothetical negative sense protein (NSP) of ~25 kDa (Zhirnov et al., 2007). However, the role of NSP still remains elusive. Another protein encoded within segment 2 (in addition to PB1 and PB1-F2) has been identified, termed N40 (Wise et al., 2009).

Morphologically, the viral particles are creased with a lipid bilayer, which is derived from the host plasma membrane. The lipid bilayer contains almost 500 spikes (each of 10–14 nm), of viral proteins hemagglutinin (HA) and neuraminidase (NA), protruded out from the envelope. These spikes appear as either rod-shaped (HA) or mushroom shaped (NA). The ratio of HA to NA generally varies from 4:1 to 5:1. The high density of HA is probable to enhance the chances for viral attachment. Additionally, matrix protein 2 (M2) is also enriched into the lipid envelope. The ratio between M2 and HA is usually about 1:10 to 1:100 (Zebedee and Lamb, 1988). Envelope viral glycoproteins (HA and NA) have stumpy kinesis within envelope. These two proteins fortify the viral structure by association with underlying matrix protein 1 (M1), the most abundant envelop viral protein. M1 is associated with the viral ribonucleoproteins (vRNPs), core of the virion and links to envelop glycoproteins to support the viral particle structurally (Schaap et al., 2012)

The vRNPs is the stable lipid-free core within the virion. The vRNP appears as flexible rods in thin sectioning of the virus. Structurally, vRNPs look like a twin-stranded helix, in which vRNPs illustrate loop at one end and a bend at other. The strand seems to be folded back on it and then coiled on itself, to form a type of twin-stranded helix (Krug and Lamb, 2001). The core of vRNPs is comprised of 8 segmented RNA coated by predominant viral protein, nucleoprotein (NP) (Coloma et al., 2009; Fournier et al., 2012). The protein coated fragments are also associated to the heterotrimeric RNA dependent RNA polymerase subunits polymerase basic 1, polymerase basic 2, and polymerase acidic (PB1, PB2, PA) which is responsible for the viral genome replication and transcription both (Klumpp et al., 1997). These polymerases are present at only 30 to 60 copies per virion (Inglis et al., 1976; Krug and Lamb, 2001; Lamb and Choppin, 1976).

## VIRAL ATTACHMENT AND ENTRANCE

Innate immunity is formed by physical barriers (e.g., mucus and collectins) and innate immune responses (Holt et al., 2008). IAV infects the host by gaining access through the nose, mouth or mucosal surfaces of the respiratory tract. To initiate an infection, IAV must get access to columnar epithelial cells of respiratory tract. Epithelial cells produce a mucus layer to protect the respiratory tract from viral infection. Mucin and sialic acid compound line the epithelial cells, which are cleaved by viral envelop glycoprotein NA. The second envelop glycoprotein HA helps the virus to attach the exposed epithelial cells by binding with sialic acid. The type of sialic acid linkage to HA glycoprotein was considered to be primary element in host tropism (Hamilton et al., 2012). Human seasonal viruses almost exclusively attach to N-acetylneuraminic (sialic) acid receptors at α-2,6 linkage, whereas avian influenza viruses predominantly bind to α-2,3 sialic acid linkage. Owing to having both α-2,3 and α-2,6 linkages, pigs and several avian species (pheasants, turkeys, quails) may act as mixing vessels and can generate reassortant viruses (Medina and Garcia-Sastre, 2011). Recently, an alternative substrate C type lectin for the binding of HA has been studied, an alternate of sialic acid attachment (Upham et al., 2010). Viral attachment to the surface of host cell induces viral uptake by endocytosis, which utilizes the clatherin and clavoline dependent mechanism (Leung et al., 2012). After endocytosis, release of viral particle is pH dependent physiological event that occurs at late lysosome (Fontana et al., 2012). The acidity (pH 4.5–5.5) of the lysosome is crucial for the uncoating and subsequent release of viral RNPs (Krug and Lamb, 2001; Krug and Lamb 2001, 2006). The low pH cleaves the association between the viral M1 and RNP complex to free the viral RNA that is then imported via interaction with the cellular importin-α/β (Flint SJ, 2003; O’Neill et al., 1995; Whittaker and Digard, 2006).

## IAV INFECTION AND INVOLVED PATTERN RECOGNITION RECEPTORS

Onset of IAV infection is very acute by triggering a cascade of immune responses and switching on almost all parts of the immune defense system. Innate immune system organizes the first line of defense against the viral infection, which is quicker in response but lacks memory and specificity. Most of the initial innate immune response is release of cytokine such as interferon α/β (IFN α/β), invasion of alveolar dendritic cells and macrophages (Achdout et al., 2003; Mandelboim et al., 2001). Acute surge of cytokine release leads to the inflammation response, which is responsible for the acute onset of the clinical symptoms. Alveolar epithelial cells have been naturally equipped with receptors to sense presence of the viral RNA and very effectively initiate cellular signaling pathways to clear viral infection. These cellular receptors are categorized to several families of pattern recognition receptors (PRRs). PRRs sense the viral RNA and activate downstream signaling pathways (Bleiblo et al., 2012). Toll like receptors (TLRs), retinoic acid inducible gene-I (RIG-I), and nucleotide-binding oligomerization domain (NOD) like receptors (NLRs) are three key families of PRRs (Pang and Iwasaki, 2011) involved in sensing viral infection.

### Toll like receptors

**Figure 1:**
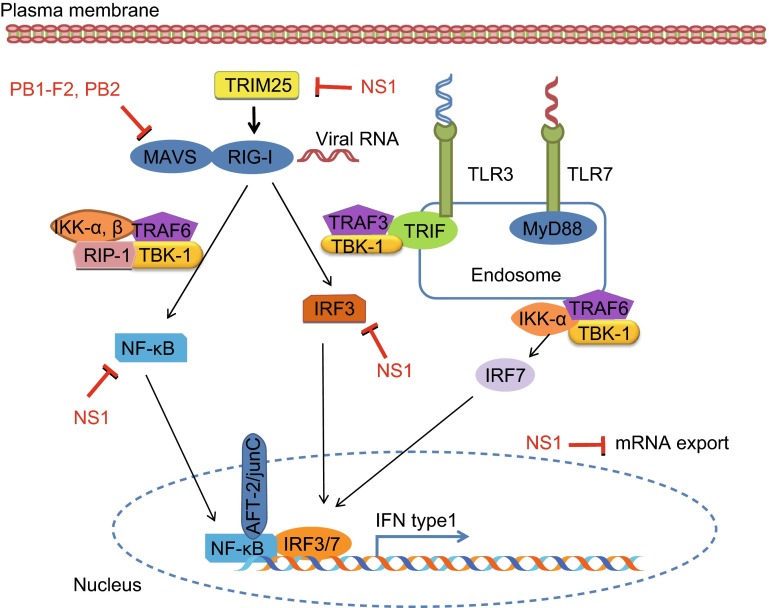
Activation of host innate immune response and function of influenza viral NS1 protein as an antagonist. The virus and its nucleic acid are sensed by the RIG-I or TLR, which leads to activation of IRF3, NF-kB, and AP-1 transcription factors. These transcription factors bind to their respective putative binding sites and initiate the transcription of type I IFN, which activates auto- or paracrine pathways to initiate the antiviral responses. However, IAV NS1 protein is able to interact with different components involved in the IFN-mediated immune responses and thereby inhibits these responses

TLRs form a major group of transmembrane receptors that are involved in the detection of viral nucleic acids (Kawai and Akira, 2011). TLRs can sense the infection of RNA viruses, TLR7 binds with the ssRNA derived from viruses like influenza virus and TLR3 senses dsRNAs (Guillot et al., 2005; Lund et al., 2004). Binding of RNA with TLRs activates the production of pro-inflammatory cytokines, chemokines, and IFN signaling pathways with the help of intracellular adaptors (Alexopoulou et al., 2001). Upon activation, TLR7 interacts with the adaptor protein myeloid differentiation factor 88 (MyD88) in plasmacytoid dendritic cells (DCs), leading to the activation of IRF7 and NF-κB that control the high levels of IFNβ and IFNα transcription (Lund et al., 2004). Similarly, TLR3 interacts with TRIF in macrophages and DCs, and TRIF activates the serine-threonine kinases IκKε (IKKε) and TBK1, resulting in phosphorylation of the transcription factor IRF3. Then IRF3 enters the nucleus and induces the expression of IFNβ (Fig. 1). Although it is well known that influenza virus activates both TLR3 and TLR7 pathways (Le Goffic et al., 2007), either activation of TLR3 or TRL7 alone could not elucidate the IFN-mediated antiviral defense during infection. In addition, TLR8 is stimulated by the ligand of ssRNA and its activation results in the production of IL-12 but not IFNα. However, the role of TLR8 in IAV infection is poorly understood (Ablasser et al., 2009). Recently, it has been shown that TLR10 may also play an important role in innate immune response to IAV infection and signaling via TLR10 is triggered by the active RNA-protein complex of influenza virus (Lee et al., 2014). However, the molecular mechanisms underlying function of these TLRs in IAV-induced innate immunity remain to be fully understood.

### RIG like receptors

RIG-I is the key receptor involved in the intracellular detection of influenza viral ssRNA during the IAV infection (Loo et al., 2008). In addition to detection of ssRNA, RIG-I and MDA5 (melanoma differentiation-associated gene 5) play a critical role by recognizing the vRNP and transcriptional intermediates containing 5′-triphosphate produced during the viral replication (Pichlmair et al., 2006). Upon sensing the viral ssRNA or transcriptional intermediates, RIG-I initiates a process that makes conformational changes to expose caspase activation and recruitment domains (CARDs). These domains are ubiquitinated by the action of E3 ligases such as TRIM25 (Munir, 2010). RIG-I can then associate with mitochondrial antiviral signaling adaptor (MAVS; also known as IPS-1, VISA or Cardif), and leads to the activation of IRF3 and NF-κB. Roles of TLRs and RIG-I receptors in the activation of IRF and NF-κB signaling have been extensively reviewed (Kawai and Akira, 2011; Loo and Gale, 2011).

### NOD like receptors

The NLRs are cytosolic pattern-recognition receptors, which form a multiprotein inflammasome complex consisting of NLRP, the adaptor ASC, and procaspase. NLRP3 is activated by membrane damage, inflammation or stress such as infection with pathogens (Martinon et al., 2009). Activation of inflammasomes results in the change of procaspase I to its active form caspase 1 by autocatalytic process (Bergsbaken et al., 2009), which contributes to production of active form of IL-1β and IL-18. NLRP3 is expressed in various cells including neutrophils, monocytes, DCs, macrophages, and human bronchial epithelial cells (Guarda et al., 2011; Pothlichet et al., 2013). Two signals are required to trigger cytokine production by the inflammasome. Signal 1 is dependent on TLR, IL-1 receptor (IL-1R), and TNFR signaling pathway, which activates the expression of the genes encoding pro-IL-1β, pro-IL-18, and NLRP3 (Martinon et al., 2009). Signal 2 is induced by host damage, which causes the activation of caspase 1 that is required for the secretion of mature IL-1β and IL-18.

Three influenza virus-associated stimuli cause the secretion of IL-1β and IL-18. First response is against ssRNA from influenza virus, which is adequate to activate the release of IL-1β from particular cell types (Thomas et al., 2009). Secondly, the influenza virus-encoded M2 ion channel in the *trans*-Golgi network causes the proton flux that can inhibit the activation of HA, which elicits NLRP3 activation and leads to the formation of the inflammasome and cleavage of pro-IL-1β and pro-IL-18 (Ichinohe et al., 2010). Third stimulus is the aggregation of influenza virus virulent protein PB1-F2 in the lysosome of lipopolysaccharide primed macrophages, leading to activation of the NLRP3 inflammasome (McAuley et al., 2013).

## ACTIVATION AND REGULATION OF HOST INNATE IMMUNE RESPONSES DURING IAV INFECTION

During inactive phase, NF-κB, IRF3/7, and c-jun/ATF-2 (AP-1) are remained in the cytoplasm. PRR sensing IAV infection causes activation of IRF3/7 through phosphorylation of the C-terminus of IRF3/7 and dimerization that leads to the exposure of nuclear localization signal (NLS), which in turn results in nuclear translocation of IRF3/7. On the other hand, the inhibitor of NF-κB (IκB) retains the NF-κB in the cytoplasm. IκB undergoes ubiquitination and proteasomal degradation upon sensing of appropriate signals by PRR. Removal of IκB leads to nuclear translocation of NF-κB via its NLS (Hayden and Ghosh, 2004). Phosphorylation of c-jun and ATF-2, two heterodimeric components of AP-1, also play their role in nuclear translocation of NF-κB. In the nucleus, these transcription factors assemble in an accommodating manner to make an enhanceosome, which binds to its respective positive regulatory domains (PRDs). The IRF3/IRF7, NF-κB, and AP-1 bind to PRD I/III, PRD II, and PRD IV, respectively, where they promote the transcription of IFN-α, IFN-β, and pro-inflammatory cytokines (TNF, IL6, IL1β, etc.). Following production, IFN-α and IFN-β mediate a positive feedback-production loop by binding to the IFN-α/β receptor (IFNAR) in an auto- and/or paracrine manner. Receptor-mediated activation of Janus kinase-signal transducer and activator of transcription (JAK-STAT) signaling results in the recruitment and phosphorylation of IRF9 into the STAT1/STAT2 heterodimer, to make interferon-stimulated gene factor 3 (ISGF3) (Mukaigawa and Nayak, 1991). Upon formation and nuclear localization of ISGF3, it binds to interferon-stimulated response elements (ISREs) (Kessler et al., 1990), which consequently leads to the transcription of hundreds of IFN stimulated genes (ISGs). These ISGs act as innate immune defense against the viral infection.

However, numerous protein phosphatases, regulatory RNA, and the suppressors of cytokine signaling (SOCS) like SOCS1 and SOCS3 are found to be involved in negative regulation of JAK-STAT pathway (Yasukawa et al., 1999). Recently, it has been shown that influenza virus-induced SOCS1 is independent of cytokine at early stages of infection both *in vitro* and *in vivo*, remarkably suppresses the activation of STAT1. This process leads to the suppression of IFN-λ-activated signaling during IAV infection. Suppression of IFN-λ response by increased level of SOCS1 activates the NF-κB signaling, which is an adaptation of host to protect cell against the viral induced SOCS1 (Wei et al., 2014). Activation of NF-κB leads to increased production of IFN-λ during IAV infection.

Long noncoding RNAs (lncRNAs) are an important class of regulatory RNAs in a variety of cellular processes. Recently, it has been shown that a human lncRNA named NRAV (Negative Regulator of Anti-Viral response) normally expressed in various human cells plays an important regulatory role in host innate immunity. Upon infection with IAV, ssRNA virus (SeV), dsRNA virus (MDRV) or DNA virus (HSV), down regulation of NRAV was observed. Further investigations revealed that NRAV is critically involved in regulation of IAV pathogenesis, as evidenced by considerably higher virulence, enhanced acute lung injury and consequently decreased survival rates of transgenic mice overexpressing the lncRNA NRAV when compared with wild type mice. Particularly, mRNA levels of some critical ISGs were substantially reduced in NRAV-overexpressing cells and transgenic (TG) mice infected with IAV, including IFIT2, IFIT3, IFITM3, OASL, and MxA. These studies suggest that NRAV is a key negative regulator of some ISGs during IAV infection *in vitro* and *in vivo*. Therefore, down regulation of NRAV in IAV-infected cells might be a host self-protection response to the virus infection, which may be critical for viral clearance (Ouyang et al., 2014). Because these ISGs play crucial roles in a wide range of immune responses, including immunity against invading pathogens, providing an antagonistic environment to limit virus propagation and spread, as well as transcriptional and translational regulation of other antiviral genes (de Veer et al., 2001), further studies are required to determine the importance of NRAV in these cellular processes.

## CELLS CRITICALLY INVOLVED IN INNATE IMMUNITY AGAINST IAV INFECTION

Infection of influenza virus in epithelial cells induces expression of many inflammatory cytokines and chemokines, such as IL-1β, IL-6, IL-8 TNF-α, CCL2, CCL3, and CXCL10 (Perrone et al., 2008). During early stages of IAV infection, CCL2 attracts alveolar macrophages and monocytes via their CCR2 receptors and activates the macrophages (Dawson et al., 2000; Herold et al., 2006; Lin et al., 2008). Once macrophages are activated during IAV infection of lungs, they produce nitric oxide synthase (NOS2) and tumor necrosis alpha (TNFα), which lead to the IAV-induced pathologic response (Jayasekera et al., 2006; Lin et al., 2008). The two discrete functions by activated macrophages upon IAV infection are very crucial for balanced responses against the viral infection. Once activated, macrophages enhance their pro-inflammatory cytokine response, including production of IFNs, IL-6, and TNF-α (Becker et al., 1991; van Riel et al., 2011). In addition, infection by influenza virus triggers activation of the alveolar macrophages that phagocytose infected cells (Kim et al., 2008). Thus, alveolar macrophages play a direct role in limiting viral spread by phagocytosis of apoptotic-infected cells and by phagocyte-mediated opsonophagocytosis of influenza virus particles (Hashimoto et al., 2007; Kim et al., 2008; Tumpey et al., 2005).

Natural killer (NK) cells are important cytotoxic and effector cells of innate immunity that serve as a crucial first-line defense against tumors and virus-infected cells. It has been previously shown that lysis of IAV-infected cells is mediated by the interaction between the NK receptors, NKp44 and PKp46, and the IAV hemagglutinin (HA) type 1 expressed by the infected cells (Mendelson et al., 2010). Cells infected with IAV express HA protein on their surface that is important for recognition by NK cells via their receptors (Mendelson et al., 2010). Association of NK cells with IAV-infected cells initiates the lysis of the target cells (Arnon et al., 2001). It has been shown that mice lacking the NKp46 receptor equivalent, NCR-1, displayed increased morbidity and mortality following influenza virus infection (Gazit et al., 2006). In addition, natural killer T (NKT) cells are known as a distinct lymphocyte lineage that regulates an expansive range of immunity. As innate immune cells, NKT cells can be activated during inflammation and viral infection. It has been suggested that NKT cells enhance the cellular immunity and regulate the pathogenesis mediated by IAV infection (Paget et al., 2011). However, many questions still remain unanswered regarding the function of NK cells and NKT cells during the IAV infection, including how these cells traffic to IAV-infected lung, their cellular dynamics, and mechanisms underlying their immunoregulatory functions.

## IAV EVASION FROM INNATE IMMUNITY

Although the host innate immune system has stout antiviral activity, it is inept in many cases to prevent influenza virus infections. This is due to the fact that influenza virus, as many other successful viruses, has evolved strategies to escape the immune response to levels that provide space for replication and transmission within their hosts. Interestingly, influenza virus uses multiple mechanisms to evade and inhibit the innate immune response of the host. On the other hand, a high immune and antiviral drug pressure in the host population pushes the influenza virus to mutate rapidly to generate new strains of IAV. The escape mechanisms by influenza viral proteins are discussed here.

### Non-structural protein 1

IAV has evolved multiple mechanisms to use its non-structural protein 1 (NS1) to mitigate the host innate immune response to influenza virus infection. NS1 is considered being the most important IFN-antagonist protein encoded by the influenza virus. This protein is highly expressed in the cytoplasm and the nucleus of infected cells where it is able to interact with different components involved in the IFN-mediated immune responses and thereby inhibits these responses (Hale et al., 2010). NS1 protein contains RNA binding domain located in first 73 amino-terminal amino acids that can bind with viral RNA and prevent it to be recognized by TLRs and RIG-I. Using reverse genetics techniques, it was observed that influenza viruses lacking or carrying the truncated NS1 gene induced increased IFN secretion from infected cells *in vivo* and *in vitro* (Ferko et al., 2004; Garcia-Sastre et al., 1998; Solorzano et al., 2005; Steel et al., 2009).

Cellular proteins like RIG-I, PKR, and OAS are activated by sensing dsRNA. However, the NS1 protein competes with these proteins for RNA binding and thereby deteriorates their activation. As OAS is less fascinating for dsRNA as compared to PKR and RIG-I, the NS1 can inhibit OAS activation precisely by binding with dsRNA (Li et al., 2006). Interestingly, NS1 protein of IAV impairs activity of RIG-I and PKR through suppressing E3 ubiquitin ligase TRIM25 that is required for posttranslational modification of RIG-I and activation of its signaling cascade including IRF-3, NF-κB, and ATF-2/c-Jun, or by direct binding with PKR (Gack et al., 2009; Gack et al., 2007; Li et al., 2006). Additionally, NS1 protein inhibits the processing of cellular mRNA in the nucleus by binding with the cellular factor CPSF30 and PABPII that are involved in transcriptional termination and polyadenylation (Chen et al., 1999; Nemeroff et al., 1998). NS1 can also interact with splicing and nuclear export factors (Satterly et al., 2007). The general inhibition of gene expression by inhibition of mRNA processing not only prevents efficient IFN expression but also suppresses the activation of ISGs. Moreover, NS1 was found to interact with a eukaryotic translation initiation factor family member eIF4B that is a key component of regulation of mRNA translation initiation. Recently, it has been revealed that influenza virus NS1 induces the degradation of the eIF4B protein (Fig. 1). Silencing of eIF4B significantly reduced the protein expression of interferon-induced transmembrane protein 3 (IFITM3), a critical protein involved in immune defense against a variety of RNA viruses via constraining the viral entry and consequently blocking the early stages of viral replication of IAV (Wang et al., 2014). Transgenic eIF4B knockdown mice infected with influenza virus showed high mortality as compared to wild type mice. It was also observed that eIF4B knockdown mice showed decreased amount of IFITM3 and high mortality by influenza virus infection compared with wild type mice (Wang et al., 2014). Besides inhibition of IFN response, NS1 plays several other roles during viral infection. However, other functions of NS1 are still unclear, suggesting it to be an exciting area of research in the near future.

### Polymerase complex

Viral proteins PB1, PB2 and PA form the influenza polymerase complex that controls the synthesis of viral RNA and mRNA. In addition, it is also involved in cap-snatching of host mRNAs and consequently reduces host cell gene expression including that of IFN-β (Dias et al., 2009). PB1 gene encodes a polypeptide of almost 80 amino acids, with specific polymorphism and has been implicated in virulence of virus with specific polymorphism (Chen et al., 2001). PB1-F2 with a serine at position 66 can interact with MAVS (Mitochondrial antiviral signaling protein), a critical mitochondrial adaptor required for IFN induction by the RLR pathway and appears to inhibit the type I IFN production (Fig. 1) (Varga et al., 2011). The 1918 pandemic influenza A/H1N1 and HPAI H5N1 are considered to have this polymorphism that is correlated with increased pathogenicity (Conenello et al., 2007).

PB1-F2 is not the only influenza viral protein that is found in the mitochondria. PB2, one of the subunits of the viral polymerase also can be found in mitochondria as well as in nucleus (Carr et al., 2006). The later can inhibit the production of type I IFN by interacting with MAVS similar to PB1-F2 (Graef et al., 2010). Interaction of PB2 with MAVS can also inhibit the IFN-β production and depends upon single amino acid polymorphism, this can be found in seasonal influenza viruses but not in highly pathogenic avian influenza (HPAI) viruses (Iwai et al., 2010). Influenza viral strains with highly efficient polymerases can evade the IFN response *in vivo* due to their frequent mutations occurred during replication (Grimm et al., 2007).

### Matrix 2 protein

**Figure 2:**
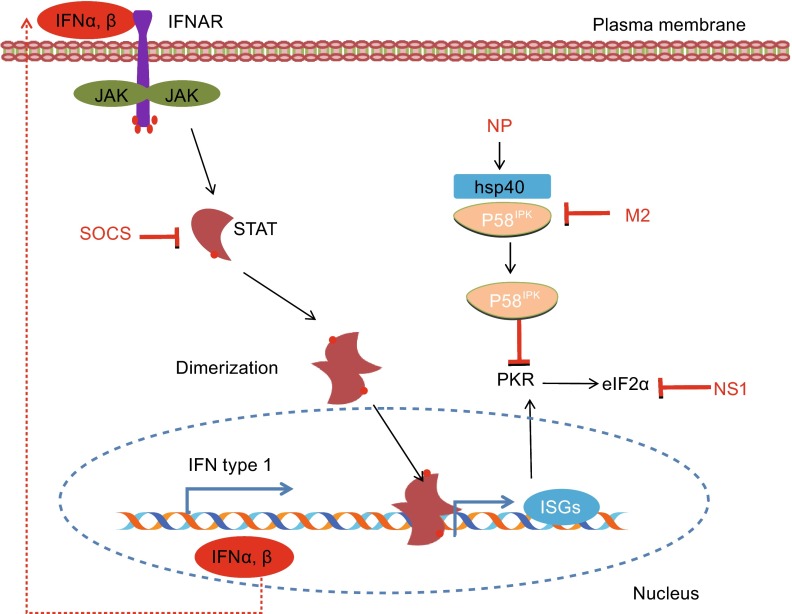
JAK-STAT signaling pathway and its interaction with viral proteins. The auto- and paracrine actions of IFNs lead to activation of JAK-STAT pathway and terminate into the formation of ISGF3. This transcription factor will initiate the transcription of hundred of interferon stimulated genes (ISGs) containing interferon stimulated response element (ISRE). IAV proteins and IAV-induced cellular proteins such as SOCS act as negative regulators of JAK-STAT signaling pathway

The M2 protein of influenza virus is a transmembrane protein that is needed for proper uncoating of influenza virus ribonucleoproteins (RNPs) during the viral entry process (Pinto et al., 1992). M2 forms a selective ion channel for the entry of viral protein. Recently, it has been observed that this protein interferes with cellular autophagy (Gannage et al., 2009). Autophagy participates in TLR activation during viral infection. Thus, it is likely that M2 is able to prevent activation of TLR. In addition, M2 protein also inhibits the P58^IPK^, a cellular regulator of PKR that forms a complex with heat shock protein 40 (hsp40) (Fig. 2), leading to the inhibition of protein translation, activation of apoptosis pathway, and escape of newly formed viral particles from host (Guan et al., 2011).

### Use of cellular proteins

Interestingly, influenza viruses not only use their own proteins to evade the innate immune system, but also take advantage of specific cellular regulators of immune system to further minimize the antiviral effects by host cells. Recently, it has been demonstrated that influenza virus-induced SOCS1 inhibits the phosphorylation of STAT1 at early stages of infection (Wei et al., 2014). Strikingly, virus-induced SOCS proteins are also involved in the inhibition of IFNs signaling (Fig. 2) (Jia et al., 2010). Influenza virus inhibits the PKR activity by interfering with cellular inhibitor of PKR. It has been found that the viral NP dissociates the P58^IPK^ from hsp40 and minimizes the antiviral state of cell (Sharma et al., 2011). In addition, influenza virus can exploit the intracellular trafficking regulator proteins, such as Rho family members, for intracellular transport of its components. For example, it has been known that infection with influenza virus significantly down regulates the ARHGAP21, an inhibitor of Cdc42 activation and thereby activates Cdc42 signaling in infected cells. Experimental studies have demonstrated that activated Cdc42 is required for efficient transport of influenza virus NA toward the plasma membranes (Wang et al., 2012). While cells with overexpression of ARHGAP21 showed reduced concentration of NA at cell surface as compared to control, disruption of ARHGAP21 expression facilitates the NA transportation to the plasma membrane (Wang et al., 2012).

## CONCLUSIONS

In last decade, our knowledge on innate immune responses against influenza infections has expanded significantly. As a result, serious efforts were made to utilize the gained knowledge for designing better vaccines and accompanying control measures. While our understanding increased on the virus-induced innate immunity, it remains to better understand the dynamics and breadth of innate immunity in tissue, species, and host-specific manners. Although more viral proteins are being identified in the influenza viruses, their importance in the influenza pathogenesis is not clearly elucidated. In particular, little information is available about their role in viral escape from host innate immunity. Bridging these gaps will pave the ways not only for designing better vaccines but also for developing novel antiviral agents.

## ABBREVIATIONS

HA: hemagglutinin
IAV: influenza A virus
IFN: interferon
M1: matrix protein 1
M2: matrix protein 2
NA: neuraminidase
NLR: NOD like receptors
NP: nucleoprotein
PA: polymerase acidic
PB1: polymerase basic 1
PB2: polymerase basic 2
PRR: pattern recognition receptor
RIG-I: retinoic acid inducible gene-I
TLR: Toll like receptors
vRNPs: viral ribonucleoproteins
